# Biological and Pathological Studies of Rosmarinic Acid as an Inhibitor of Hemorrhagic *Trimeresurus flavoviridis* (habu) Venom

**DOI:** 10.3390/toxins2102478

**Published:** 2010-10-25

**Authors:** Hnin Thanda Aung, Toshiaki Nikai, Yumiko Komori, Tsunemasa Nonogaki, Masatake Niwa, Yoshiaki Takaya

**Affiliations:** 1 Faculty of Pharmacy, Meijo University, 150 Yagotoyama, Tempaku, Nagoya, 468-8503, Japan; Email: hninthandaaung07@gmail.com (H.T.A.); nikai@meijo-u.ac.jp (T.N.); ykomori@meijo-u.ac.jp (Y.K.); masa@meijo-u.ac.jp (M.N.); 2 College of Pharmacy, Kinjo Gakuin University, 2-1723 Omori, Moriyama, Nagoya, 463-8521, Japan; Email: tunenono@kinjo-u.ac.jp

**Keywords:** rosmarinic acid, snake venom, hemorrhage, metalloproteinase, *Argusia argentea*

## Abstract

In our previous report, rosmarinic acid (RA) was revealed to be an antidote active compound in *Argusia argentea* (family: Boraginaceae). The plant is locally used in Okinawa in Japan as an antidote for poisoning from snake venom, *Trimeresurus flavoviridis* (habu). This article presents mechanistic evidence of RA’s neutralization of the hemorrhagic effects of snake venom. Anti-hemorrhagic activity was assayed by using several kinds of snake venom. Inhibition against fibrinogen hydrolytic and collagen hydrolytic activities of *T. flavoviridis* venom were examined by SDS-PAGE. A histopathological study was done by microscopy after administration of venom in the presence or absence of RA. RA was found to markedly neutralize venom-induced hemorrhage, fibrinogenolysis, cytotoxicity and digestion of type IV collagen activity. Moreover, RA inhibited both hemorrhage and neutrophil infiltrations caused by *T. flavoviridis* venom in pathology sections. These results demonstrate that RA inhibited most of the hemorrhage effects of venom. These findings indicate that rosmarinic acid can be expected to provide therapeutic benefits in neutralization of snake venom accompanied by heat stability.

## 1. Introduction

Envenomation resulting from snakebites is an important public health hazard in many regions, particularly in tropical and subtropical regions [1,2,3]. Snake envenomation causes various pathophysiological changes such as inflammation, increased body temperature, hemorrhage, necrosis, nephrotoxicity, cardiotoxicity, haemostatic changes and ultimately death [4]. Hemorrhage is a common symptom associated with local tissue damage in snake poisoning by Viperidae. In severe poisoning, hemorrhage can be observed in many internal organs [5,6]. Hemorrhage can occur as a result of cytotoxicity of the venom on endothelial cells, and a degradation of the basement membrane of the vein due to the venom acting as a metalloproteinase. In many countries, plant extracts have been traditionally used in the treatment of snakebite envenomations [7,8,9], although only a few cases have been scientifically validated. The present study aims to examine the venom neutralization potential of rosmarinic acid (RA) isolated from a methanolic extract of *Argusia argentea* [10] and its mode of action.

## 2. Materials and Methods

### 2.1. Materials

*Trimeresurus flavoviridis* (habu) venom (Okinawa), *Gloydius blomhoffii* venom and *Bitis arietans* venom were purchased from Japan Snake Institute, Gunma. *Crotalus atrox* venom was purchased from Sigma-Aldrich. Hemorrhagic toxin b (HT*b*) from *C. atrox* venom was prepared by the method reported previously [11]. Bilitoxin-2 and Ac_1_-proteinase were isolated using our methods reported previously [12,13] for *Agkistrodon bilineatus* venom and *Deinagkistrodon acutus* venom, respectively. Hemorrhagic toxin-1 (HT-1) was obtained from *B. arietans* venom [14]. Human and bovine fibrinogens were supplied by Sigma-Aldrich, Tokyo, Japan. Type IV collagen was purchased from Nitta Gelatin Inc. Cryo-preserved human umbilical vein endothelial cells (HUVEC), its respective cell culture media (HuMedia EB-2), other cell culture supplements, and reagents were obtained from Kurabo (Osaka, Japan). The cell counting kit was purchased from Dojindo (Kumamoto, Japan). Other chemicals were of analytical grade from commercial sources. All experiments involving the use of animals were carried out in compliance with the guidelines for animal experiments of Faculty of Pharmacy, Meijo University.

### 2.2. Rosmarinic Acid

Rosmarinic acid (RA) was isolated and purified from methanolic extract of *Argusia argentea* as reported previously [10]. A voucher sample has been deposited in the Herbarium of the Faculty of Pharmacy, Meijo University, Japan.

### 2.3. Anti-hemorrhagic Activity Assay

Anti-hemorrhagic activity was assayed by the method of Bjarnason and Tu [15] using ddY mice of 20 g average weight. Two groups of four mice were used for the experiment. All crude venom solutions of *T. flavoviridis* venom, *C. atrox* venom, *G. blomhoffii* venom and *B. arietans* venom, were prepared at a concentration of 0.14 mg/mL in saline. Concentrations of purified hemorrhagic toxin solutions were as follows: HT*b* (0.41 mg/mL), bilitoxin-2 (2.75 μg/mL), HT-1 (0.29 mg/mL), and Ac_1_‑proteinase (1.04 mg/mL). A test solution was prepared by mixing the venom solution or the toxin solution (50 µL) and RA (0.5 mg/mL in 10% DMSO-saline, 50 µL) followed by 10 min incubation at 37 °C. These test solutions (100 µL) were injected subcutaneously (s.c.) in the abdomen of mice. Similarly, a group of mice which were injected with a venom solution without RA was used as a control group, and also a group which was only injected with 10% DMSO-saline (50 μL) served as a blank group. Prior to this study, effects of DMSO at several concentrations were investigated, and DMSO at less than 10% was found to cause no significant inactivation of venom. After 24 h, mice were euthanized by inhalation of chloroform, the skin covering the abdomen was removed and hemorrhagic lesions were analyzed as follows. The area of the lesion was estimated by major and minor axes measurements, since the shape of the lesions are always amorphous, like an ellipse. 

### 2.4. Fibrinogen Hydrolytic Activity Assay

Fibrinogen hydrolytic activity was assayed by the method of Ouyang and Teng [16]. A solution of 0.1% human fibrinogen in 50 mM Tris-HCl buffer (pH 7.5) (1 mL) and a venom solution (50 µL of 0.21 mg/mL of *T. flavoviridis* venom or 5.5 µg/mL of bilitoxin-2) were incubated in the presence or absence of RA (0.5 mg/mL) at 37 °C. At various time intervals, aliquots of 100 µL of denaturing solution (10 mM phosphate buffer, pH 7.2, containing 10 M urea, 4% sodium dodecyl sulfate (SDS), and 4% β-mercaptoethanol) were added. This solution was incubated at 37 °C for 6 h and then run on 10% polyacrylamide slab gel electrophoresis. Electrophoresis was carried out for 2 h with a current of 25 mA per slab gel. Bromophenol blue (BPB) solution was used as an indicator.

### 2.5. Collagen Hydrolytic Activity Assay

Collagen hydrolytic activity was assayed as follows. Sodium hydrogen carbonate (60 µL, pH 12) was added to 0.3% type IV collagen (0.9 mL) and adjusted to pH 8. Aliquots of type IV collagen were incubated with *T. flavoviridis* venom (0.21 μg/mL) in the presence or absence of RA (0.5 mg/mL). At various time intervals, aliquots of 100 µL of denaturing solution (10 mM phosphate buffer, pH 7.2, containing 10 M urea, 4% SDS, and 4% β-mercaptoethanol) were added. This solution was boiled for 3 min and run on SDS-PAGE using a 7.5% polyacrylamide slab gel electrophoresis.

### 2.6. Cytotoxic Action on HUVEC

The effects of RA and *T. flavoviridis* venom on cultured human umbilical vein endothelial cells (HUVEC) were investigated [17,18,19]. Frozen HUVEC were cultured and maintained in commercially available media, HuMedia-EB2, supplemented with fetal calf serum (2% v/v), hEGF (10 ng/mL), hFGF-B (5 ng/mL), hydrocortisone (1 µg/mL), heparin (10 µg/mL), gentamicin (50 µg/mL), and amphotericin B (50 ng/mL). At confluency, cells were trypsinized, washed with the same medium and then resuspended in growth media. These cells were seeded in 96-multiwell plates (5 × 10^3^ cells per well in 100 µL medium) and were allowed to attach and reach log phase of growth. Aliquots of venom and RA to be assayed were diluted in saline and were sterilized by filtration with cellulose acetate 0.22 µm membrane filters. Various concentrations of RA (0.5, 0.25, 0.125, 0.06, 0.03 mg/mL in 10% DMSO-saline) in the presence or absence of *T. flavoviridis* venom (0.14 mg/mL) were added to each well in 100 µL medium. The plate was incubated at 37 °C under 5% CO_2_ atmosphere for 17 h. Ten microliters of cell counting kit-8 was added to each well, and the microplate was incubated for 1 h, after which cell densities were measured at 450 nm using Bio-RAD Model 550 MicroplateR eader.

### 2.7. Histopathological Study

Histopathological study for RA was performed by intramuscular (i.m.) injection of *T. flavoviridis* venom solution into the medial aspect of the thigh muscle of ddY strain white mice. Histopathological study of muscle was conducted in three groups. Group A was injected with the venom (0.21 mg/mL, 100 µL), while group B was injected with RA (0.5 mg/mL, 100 µL). Group C was injected with a mixture of the venom (0.41 mg/mL, 50 µL) and RA (0.25 mg/mL, 50 µL). Test solutions were preincubated at 37 °C for 10 min before injection. The mice were killed by chloroform inhalation 24 h after injection. Tissue samples were immediately fixed in buffered formate fixative for 24 h at room temperature. The tissue was then washed for 4 h in running water, dehydrated in an autotechnicon, and stained with hematoxylin and eosin for observation under light microscope.

### 2.8. Heat Stability

RA (0.5 mg/mL) in 10% DMSO-saline was heated at 37 °C, 50 °C and 100 °C for 10 min, respectively. Fifty microliters of each heat-treated RA solution was mixed with *T. flavoviridis* venom (0.21 mg/mL, 50 µL) and incubated at 37 °C for 10 min. An aliquot of 0.1% human fibrinogen in 50 mM Tris-HCl buffer, pH 7.5 was added to each test tube. The reactions were stopped by adding 100 µL of denaturing solution (10 mM phosphate buffer, pH 7.2, containing 10 M urea, 4% SDS, and 4% β-mercaptoethanol). These solutions were incubated at 37 °C for 6 h and then run on 10% polyacrylamide slab gel electrophoresis. The venom solution without a sample was also subjected to SDS-PAGE for comparison. Moreover, by using RA (0.5 mg/mL), which was treated at 100 °C, antihemorrhage activity was also examined as mentioned above.

### 2.9. Assay for Edema Activity

Hind-paw edema activity was assayed by the method of Ho *et al*. [20]. Four ddY strain white mice (20–23 g) were individually injected in the right foot pad with *Trimeresurus elegans* venom (12.5 µg in 50 µL of 10% DMSO-saline). An equal volume of 10% DMSO saline was injected into the left paws as control. Inhibition assays were performed by preincubated rosmarinic acid (0.5 mg/mL in 10% DMSO saline) with toxin for 10 min at 37 °C. The volume of each paw was measured with a slide caliper. The degree of paw swelling was expressed as % increase of the initial paw volume.

## 3. Results

### 3.1. Inhibitory Activity of RA on Crude Snake Venoms and Purified Hemorrhagic Toxins

**Figure 1:**
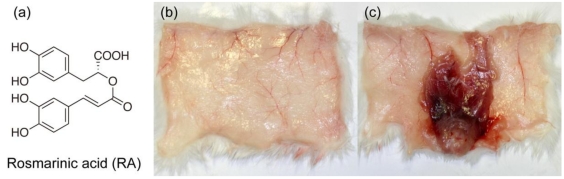
Inhibitory activity of RA on *T. flavoviridis* venom (**a**) Structure of rosmarinic acid, (**b**) *T. flavoviridis* venom with RA, (**c**) *T. flavoviridis* venom without RA.

When crude venom (*T. flavoviridis* (habu) venom, *C. atrox* venom, *G. blomhoffii* venom, and *B. arietans* venom) or purified toxin, (HT*b*, bilitoxin-2, HT-1 and Ac_1_-proteinase) was injected s.c. in the abdomen of mice, a distinct hemorrhagic lesion was observed (Figure 1c) [10]. No hemorrhagic spots were produced after s.c. injection of crude venom or purified toxin with RA (Figure 1b). RA effectively inhibited the hemorrhagic activities of crude venoms as well as purified hemorrhagic toxins.

### 3.2. Inhibition of Fibrinogen Hydrolytic Activity

**Figure 2:**
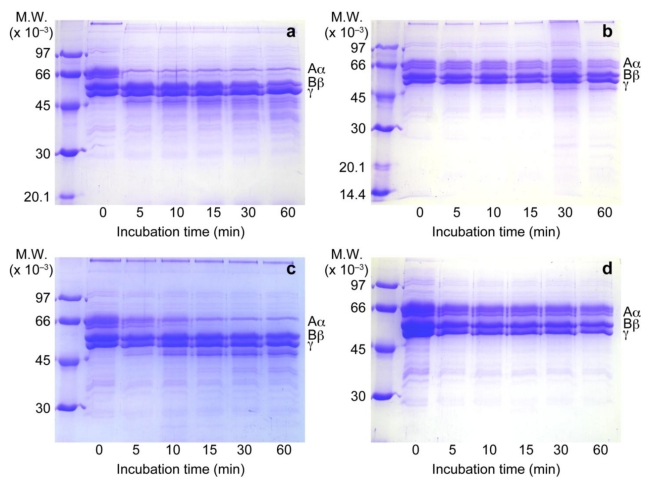
Effect of RA on human fibrinogen hydrolytic activities by *T. flavoviridis* venom and bilitoxin-2. 10% SDS-PAGE of time-dependent digestion of human fibrinogen by *T. flavoviridis* venom and bilitoxin-2 in the presence or absence of RA. (**a**) *T. flavoviridis* venom without RA; (**b**) *T. flavoviridis* venom with RA; (**c**) bilitoxin-2 without RA; (**d**) bilitoxin-2 with RA. Molecular weight makers of 97, 66, 43, 30, 20.1, and 14.4 kDa were used.

When human fibrinogen was incubated with *T. flavoviridis* venom, the Aα band of the fibrinogen disappeared on SDS-PAGE, whereas the Bβ chain and γ chain were essentially unaffected (Figure 2a). The venom with RA did not reveal any apparent degradation of human fibrinogen (Figure 2b). RA also inhibited Aα hydrolysis by bilitoxin-2 (Figure 2c and 2d).

### 3.3. Inhibition of Venom Cytotoxic Action on HUVEC

**Figure 3:**
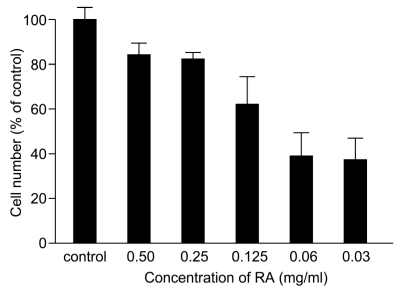
Effects of RA against the cytotoxic actions of *T. flavoviridis* venom on HUVEC.

RA alone had no effect on the viability of HUVEC, but it markedly protected HUVEC from the toxic effects of *T. flavoviridis* venom (0.14 mg/mL) at all concentrations of RA tested (0.50, 0.25, 0.125, 0.06, and 0.03 mg/mL) (Figure 3). The maximum (84.2%) protective effect of RA was exhibited with RA at 0.5 mg/mL.

### 3.4. Inhibition of Type IV Collagen Hydrolytic Activity

**Figure 4:**
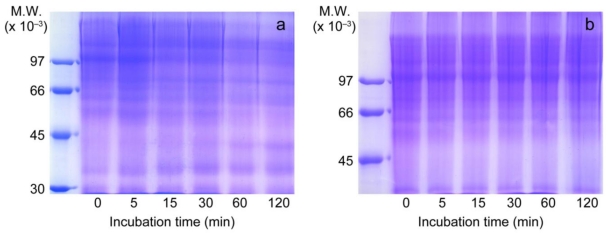
Effect of RA on type IV collagen hydrolytic activity by *T. flavoviridis* venom. 7.5% SDS-PAGE of time-dependent digestion of type IV collagen by *T. flavoviridis* venom in the presence or absence of RA. (**a**) venom without RA, (**b**) venom with RA. Molecular weight makers of 97, 66, 43, and 30 kDa were used.

Type IV collagen was incubated with *T. flavoviridis* venom for different periods of time. The venom completely degraded type IV collagen (104 kDa), especially over 1 h, and degradates with smaller molecular weights (43 and 35 kDa) appeared, as shown in Figure 4a. In the presence of RA (0.5 mg/mL), type IV collagen was not digested by incubation with the venom (Figure 4b).

### 3.5. Histological Study of T. flavoviridis Venom and the Effect of RA

**Figure 5:**
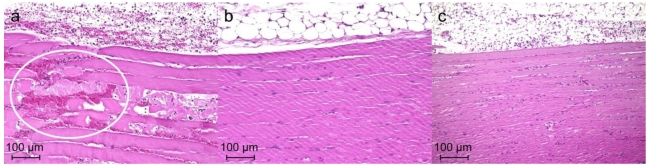
Histological results of thigh muscle after (**a**) injection of *T. flavoviridis* venom (0.21 mg/mL) alone (**b**) injection of RA (0.5 mg/mL) alone (normal muscle), (**c**) injection of a mixture of RA (0.25 mg/mL) and the venom (0.41 mg/mL).

Both hemorrhage and neutrophil infiltrations were observed in a wide area (of the circle) after injection of *T. flavoviridis* venom (0.21 mg/mL) (Figure 5a). The result showed normal musculature devoid of hemorrhage and neutrophils in the muscle fibers after injection of RA (0.5 mg/mL) (Figure 5b). There was no hemorrhage or neutrolphil infiltration in the muscle fibers after injection of a mixture of the venom (0.41 mg/mL) and RA (0.5 mg/mL, or 0.25 mg/mL; Figure 5c).

### 3.6. Heat Stability of RA

**Figure 6:**
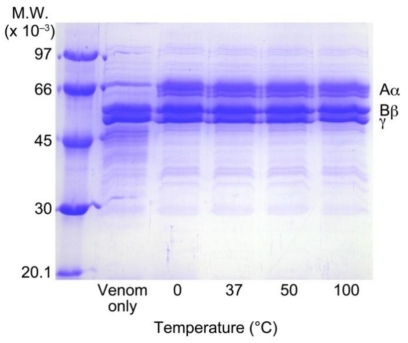
Heat stability study of RA by human fibrinogen digestion by *T. flavoviridis* venom. RA was heated at 37 ºC, 50 ºC, or 100 ºC, then RA (0.5 mg/mL) was mixed with human fibrinogen and venom (0.21 mg/mL), and the solution was incubated at 37 °C for 10 min. Aliquots were subjected to SDS-PAGE (7.5% gel).

RA, which was heated at various temperatures, was incubated with human fibrinogen and *T. flavoviridis* venom. RA inhibited the digestion of Aα chain of human fibrinogen after treatment at each temperature (Figure 6). RA (0.5 mg/mL), which was heated at 100 °C for 10 min, also showed complete inhibition against *T. flavoviridis* venom.

### 3.7. Inhibition of Venom-Induced Edema

The edema-forming activity was assayed using four mice. *T. elegans* venom induced an edema of 30% in the mouse footpad, at a dose of 12.5 µg. When *T. elegans* venom was preincubated with RA (0.5 mg/mL), the edema-forming was significantly reduced.

## 4. Discussion

**Figure 7:**
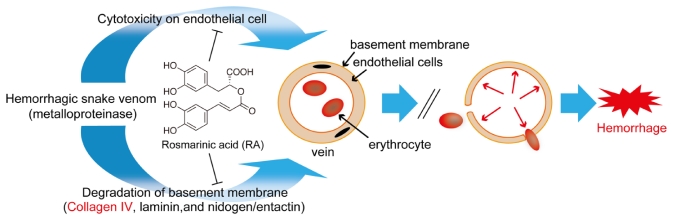
Mechanisms of hemorrhage induction by snake venom, and points of action of RA for inhibition of hemorrhage.

RA effectively inhibited snake venom induced hemorrhage by crude venoms of *T. flavoviridis*, *Crotalus atrox, Gloydius blomhoffii*, and *B. arietans* or purified toxins (HTb, bilitoxin-2, HT-1 and Ac_1_-proteinase) [10]. As shown in Figure 7, envenomation by snakebites often produces persistent hemorrhage due to considerable degradation of fibrinogen and other coagulation factors, thus preventing clot formation [21]. The pathogenesis of venom-induced hemorrhage involves direct damage to endothelial cells in microvessels by hemorrhagic toxins [22,23]. Snake venom metalloproteinases (especially snake venom metalloproteinase from *T. flavoviridis* venom) degrade the most important components of the basement membrane, such as laminin, type IV collagen and nidogen/entactin [24,25,26]. In this study, an attempt was made to determine the protective effects of RA on digestion of human fibrinogen, digestion of type IV collagen and cytotoxic action on HUVEC induced by *T. flavoviridis* venom. The pure compound showed antifibrinogenolytic activity by inhibiting the digestion of the Aα chain of human fibrinogen. RA also effectively inhibited HUVEC against the toxic action of *T. flavoviridis* venom at various concentrations and digestion of type IV collagen. Moreover, the pathological study of thigh muscles showed that RA inhibited hemorrhage and neutrophil infiltrations. *T. flavoviridis* venom-induced lethality was significantly antagonized by RA (960 µg), whereas the venom is highly lethal to mice with 200 µg. The compound inhibited the edema‑forming effect of *T. elegans* venom and lethal action induced by *T. flavoviridis* venom. The aforementioned evidence demonstrates that RA inhibited most of the hemorrhage effects of venom (Figure 7). It has been reported that RA isolated from *Cordia verbenacea* (Boraginaceae) inhibits the edema and myotoxic activity induced by crude venom and isolated PLA_2_s [27]. However, the mechanisms of action of PLA_2_s are quite different from those of metalloproteinases. This is the first report of RA that demonstrates the inhibitory mechanism for snake venom induced hemorrhage and protection from snakebite envenomation.

In this study, the inhibitory activities of rosmarinic acid from *Argusia argentea* against the action of snake venom were investigated. The plant is found on tropical shores of the Pacific and Indian oceans and the extract of the leaves are locally used as an antitoxin for snakebites and fish poisoning [28,29]. In the Okinawa islands, the leaves of *Argusia argentea* are the first medical treatment against snake venom as well as against jellyfish venom [30]. Hemorrhage is a conspicuous sequela of envenomation by Viperidae. Hemorrhage from damage to the vascular endothelium of vital organs causes death [31]. These observations confirmed that RA possesses potent snake venom neutralizing properties. Furthermore, the heat stability of RA would satisfy a requirement for a first-aid treatment for snakebite. Because snakebites often occur outdoors far from medical institutions, it is necessary to distribute a drug to distant locations without refrigeration, and the drug must be storable at room temperature. Further studies are needed to investigate post-administration of RA, and the effects of different methods of administration. After these issues are resolved, RA could become a potent alternative antidote compound for snake envenomation.
